# Supplementary material for: The Berlin Misophonia Questionnaire Revised (BMQ-R): Development and validation of a symptom-oriented diagnostical instrument for the measurement of misophonia
Source: PLoS One. 2022 Jun 21;17(6):e0269428. doi: 10.1371/journal.pone.0269428 (PMC9212156; doi:10.1371/journal.pone.0269428)
Supplement: S1 Text — (DOCX) [file pone.0269428.s001.docx]

Pretest of the Berlin Misophonia Questionnaire (BMQ) main study

Nico Remmert^1*^, Katharina Maria Beate Schmidt^1^, Patrick Mussel^1^, and Michael Eid^1^

^1^ Department of Psychology, Freie Universität Berlin, Berlin, Germany

^*^ Corresponding author

E-mail: n.remmert@fu-berlin.de

**Pretest of the Berlin Misophonia Questionnaire (BMQ) main study**

The pretest had two objectives: 1) to scrutinize the translated scales regarding their reliability and factorial validity 2) to examine interrater reliability and agreement of the revised sound classes of the Berlin Misophonia Questionnaire (BMQ-R).

**Translation procedure**

Permission to translate the S-Five-E (Vitoratou et al. 2020), MisoQuest (Siepsiak et al., 2020), STICSA (Ree et al., 2008), and DPSS (Cavanagh & Davey, 2000) was obtained from the original test authors, respectively. Scales were translated using the TRAPD procedure (Harkness, 2003). TRAPD is an acronym for several steps needed to produce high-quality translations of questionnaires, namely translation, review, adjudication, pretesting, and documentation (Harkness, 2003). For each scale, at least two independent, fluent, or native speakers of the respective language (Polish or English) as well as German translated the items to German. The translated versions were then reviewed with the translators and the authors of this study regarding the intended content, wording, and authenticity of the items. If necessary, alterations were implemented. In addition to the original TRAPD procedure, a backtranslation loop was incorporated to assure that the test authors approved the translated version. The backtranslation was done by a separate translator for each language, respectively. Again, alterations were implemented if necessary. The final translated versions were then scrutinized in the pretest.

**Revised Sound Classes**

To assess the quality of the revised sound classes (i.e., groups of sounds which share subjective meaning or specific features), participants assigned four specific sounds from each of the 12 revised sound classes (hence, 48 sounds in total). Definitions of the sound classes can be found in Table 1. The specific sounds were randomly chosen from the misophonic trigger list by Potthast et al. (2018). To balance the number of specific sounds per class, the most frequently mentioned sounds from Remmert (2017) were chosen to replenish classes with an insufficient number of sounds. The additional sound class “internal body sounds” was not tested because these sounds do not occur in the misophonic trigger list and the class only comprises very few sounds. Interrater reliability was evaluated by means of Krippendorff’s α (K.α; Krippendorff, 1980), Conger’s κ (κ; Conger, 1980), and a simple percentage agreement without tolerance. Further, the relative frequency of the intended classifications was calculated.

**Model specification, model fit evaluation, and item selection procedure**

In line with the measurement models proposed by the authors of the original scales, confirmatory factor analysis (CFA) models were specified. All models were estimated through maximum likelihood estimation with robust (Huber-White) standard errors (Huber, 1967; White, 1980) and a scaled test statistic to address nonnormality of the data. Full information maximum likelihood estimation was applied to take account of missing responses. The R package “lavaan” (version 0.6-9; Rosseel, 2012) was used for all model specifications.

Model fit was evaluated using the χ²-test and common standards of fit index cutoffs (RMSEA close to .06, SRMR close to .08, Mc (McDonald’s Centrality) close to .90; Hu & Bentler, 1999) as well as a CFI (Comparative Fit Index) close to .97, and a TLI (Tucker-Lewis Index) close to .97 (Schermelleh-Engel et al., 2003). Moreover, AIC (Akaike Information Criterion), BIC (Bayesian Information Criterion), and ECVI (Expected Cross Validation Index) were computed to compare non-nested models.

When sufficient model fit could not be achieved, items were selected by a brute-force algorithm implemented in the R package “stuart” (version 0.9.1-9000, Schultze, 2019). The algorithm evaluates the model fit of every possible item combination to obtain the best combinatorial and psychometric solution according to a defined model fit criteria function (Schultze, 2017). An adjusted function optimizing the CFI, RMSEA, and SRMR was used.

**Participants**

Bandalos (2014) suggested a required sample size between 200 and 500 for CFAs with ordinal data using a Maximum Likelihood Robust (MLR) estimator. Consequently, we decided to collect a sample greater than 200 participants resulting in a total of *N* = 241. Participants were recruited in Germany via online posts on social media (Facebook and Instagram) and university mailing lists. Specific groups of individuals identifying as having misophonia as well as unspecific groups were chosen. Psychology students received course credit as an incentive. For subsequent analyses, a total of *N* = 236 participants with less than 50% missing responses and a response pattern index^[[1]](#footnote-1)^ (Meade & Craig, 2012) less than 18% of the total items of the respective instrument were included. Almost all participants were female (89.0%), and the mean age was *M* = 34.92 years (*SD* = 11.17, range 16 to 69). Further, most of the participants (67.0%) had at least a college entrance qualification, whereas only 12.7% were students. More than half of the participants (56.8%) were single and 31.8% were married. Almost a quarter of the sample was either part-time or marginally employed, whereas 51.3% were full-time employed.

**Results**

Model fit indices of the specified CFAs on the translated scales are given in Table 2. The Disgust Propensity model showed excellent model fit exceeding all specified cutoff criteria. The estimated internal consistency was ω _=_  .75, which is comparable with the original scale (α = .83; Fergus & Valentiner, 2009). For reliability estimates (internal consistency) of the pretest scales see Table 3. No alterations regarding the usage of the scale in the main study were indicated.

For the *S-Five*, the model did not show adequate fit to the data according to the specified cutoffs. However, the RMSEA and the SRMR indicated acceptable fit. To maintain the original structure, no alterations were intended for the main study. Reliability estimates (.82 ≤ ω ≤ .90) were in line with the original scales.

The unidimensional 14-item model of the translated *MisoQuest* did not show adequate fit to the data. To retain as much items as possible, only two items were excluded after conducting a brute force optimization procedure. Further, it was assured to preserve the content areas of the questionnaire. The 12-item optimized version showed acceptable fit which was better than for the 14-item version (ΔAIC = 1088.80, ΔBIC = 1109.58, ΔECVI = 0.41). The internal consistency was estimated at ω = .87 being lower than for the original scale (α = .96).

Regarding the *STICSA*, the model did not show adequate fit to the data according to the specified cutoffs. A brute force optimization procedure yielded an 18-item version discarding 3 items. We thoroughly checked the loss of content of the remaining items to keep it as marginal as possible. This optimized version showed excellent fit to the data and had a better fit than the 21-item version (ΔAIC = 1670.64, ΔBIC = 1701.59, ΔECVI = 0.84). Regarding reliability estimates, we found similar results for the cognitive anxiety dimension (ω = .84) as well as for the somatic anxiety dimension (ω = .82) compared to the original scales (α = .88 for both scales).

**Revised Sound Classes**

Interrater reliability and agreement estimates for the assigned sounds are given in Table 4. Prior to analyses, the sounds were grouped in line with their respective sound class. Therefore, interrater reliability was assessed for the sounds within their matched class, respectively. None of the assigned sound groups exceeded the proposed minimum of K.α ≥ .67 to draw further conclusions (Krippendorff, 2004). Regarding Conger’s κ, the interrater reliability of the sounds ranged from poor to fair according to Landis and Koch (1977). Considering the simple percentage of agreement without tolerance, interrater agreement was provided for some sounds (“people eating,” “human voices,” “external environment sounds,” “finger and hand sounds,” and “animal sounds”). However, some sounds were not rated similarly to a sufficient extent.

The relative frequency of intended classifications takes another qualitative facet into account, apart from the interrater reliability of the sounds. For almost each sound group more than half of the participants chose the intended sound class, except for the class “rustling, scratching, and squeaking.“

Conclusively, interrater reliability is not sufficient, so ratings are dependent on raters. Sounds cannot be reliably assigned to the proposed sound classes. This may be due to the given sounds, defined sound classes, or raters. On the one hand, sounds might be non-representative for the sound classes which impedes agreement between raters. On the other hand, the sound classes might be overlapping and therefore impede the assignment as a specific sound might fit more than one sound class. Another possible explanation might be the properties of the raters. Raters were only briefly instructed which might have cause disagreement since the sound classes were not fully comprehended. Further, participants had to rate every of the 48 sounds which might have caused exhaustion effects producing nuisance. The large number of sound classes (12) might have been further nuisance. Moreover, literature has shown that sounds are chiefly dependent on the subjective meaning (Brout et al., 2018; Jastreboff & Jastreboff, 2014) and on the contextual information involved (Edelstein et al., 2020). This may cause a low reliability when rating sounds by classes. However, a large proportion of the sounds were assigned to their respective intended sound class. The findings suggest that individuals cannot consistently assign sounds to their respective sound class. However, most of the participants assigned sounds to their intended sound class.

We suggest to apply principal component analyses or cluster analyses for ratings on specific sounds (e.g., the misophonic trigger list by Potthast et al. (2018); or the S-Five-t triggers check list by Vitoratou et al. (2020)). We believe that this elucidates commonalities between sounds more rigorously, and thus may help to find less overlapping and more reliable sound classes.

**Acknowledgement**

We thank Kevin Sturm, Maik Hante, Rebecca Gruzman, Gloria Gierlach, Friedemann Trutzenberg, and Nicole Chantal Skerstupeit for their translations of the validation scales.

**References**

Bandalos, D. L. (2014). Relative Performance of Categorical Diagonally Weighted Least Squares and Robust Maximum Likelihood Estimation. *Structural Equation Modeling: A Multidisciplinary Journal*, *21*(1), 102-116. <https://doi.org/10.1080/10705511.2014.859510>

Brout, J. J., Edelstein, M., Erfanian, M., Mannino, M., Miller, L. J., Rouw, R., Kumar, S., & Rosenthal, M. Z. (2018). Investigating Misophonia: A Review of the Empirical Literature, Clinical Implications, and a Research Agenda. *Frontiers in Neuroscience, 12*(36). <https://doi.org/10.3389/fnins.2018.00036>

Cavanagh, K., & Davey, G. C. L. (2000). *The development of a measure of individual differences in disgust* [Paper presentation]. British Psychology Society, Winchester, UK.

Conger, A. (1980). Integration and generalization of kappas for multiple raters. *Psychological Bulletin*, *88*(2), 322–328. <https://doi.org/10.1037/0033-2909.88.2.322>

Edelstein, M., Monk, B., Ramachandran, V. S., & Rouw, R. (2020). *Context influences how individuals with misophonia respond to sounds*. bioRxiv. <https://doi.org/10.1101/2020.09.12.292391>

Fergus, T. A., & Valentiner, D. P. (2009). The Disgust Propensity and Sensitivity Scale–Revised: An examination of a reduced-item version. *Journal of Anxiety Disorders*, *23*(5), 703-710. <https://doi.org/10.1016/j.janxdis.2009.02.009>

Harkness, J. (2003). Questionnaire Translation. In J. Harkness, F. van de Vijver, & P. Mohler (Eds.), *Cross-cultural survey methods* (pp. 35–56). Wiley.

Hu, L., & Bentler, P.M. (1999). Cutoff criteria for fit indexes in covariance structure analysis: Conventional criteria versus new alternatives. *Structural Equation Modeling, 6*(1), 1-55. <https://doi.org/10.1080/10705519909540118>

Huber, P. J. (1967). The Behavior of Maximum Likelihood Estimates under Nonstandard Conditions. In L. M. Le Cam & J. Neyman (Eds.), *Proceedings of the Fifth Berkeley Symposium on Mathematical Statistics and Probability* (Vol. 5, pp. 221-233).

Jastreboff, P. J., & Jastreboff, M. M. (2014). Treatments for Decreased Sound Tolerance (Hyperacusis and Misophonia). *Seminars in Hearing, 35*(2), 105-120. <http://dx.doi.org/10.1055/s-0034-1372527>

Krippendorff, K. (1980). *Content analysis: An introduction to its methodology* (1st ed.). Sage.

Krippendorff, K. (2004). *Content analysis: An introduction to its methodology* (2nd ed.). Sage.

Landis, J. R., & Koch, G. G. (1977). The measurement of observer agreement for categorical data. *Biometrics*, *33*(1), 159-174. <https://doi.org/10.2307/2529310>

McDonald, R. P. (1999). *Test theory: A unified treatment*. Lawrence Erlbaum Associates Publishers.

Meade, A. W., & Craig, S. B. (2012). Identifying careless responses in survey data. *Psychological Methods*, *17*(3), 437-455. <https://doi.org/10.1037/a0028085>

Potthast, N., Illies, L., & Kley, H. (2018). *Misophonie Triggerliste (MT)* [Unpublished manuscript]. Department of Psychology. Universität Bielefeld.

Ree, M. J., French, D., MacLeod, C., & Locke, V. (2008). Distinguishing Cognitive and Somatic Dimensions of State and Trait anxiety: Development and validation of the State-Trait Inventory for Cognitive and Somatic Anxiety (STICSA). *Behavioural and Cognitive Psychotherapy, 36*(3), 313–332. <https://doi.org/10.1017/S1352465808004232>

Remmert, N. (2017). *Konstruktion und Validierung des Berliner Misophonie Fragebogens (BMF) zur Messung der Symptome auditiver Intoleranz distinkter Geräusche in der klinisch-psychologischen Forschung* [Bachelor’s thesis]. Freie Universität Berlin. https://osf.io/9vfms/files/

Rosseel, Y. (2012). lavaan: An R Package for Structural Equation Modeling. *Journal of Statistical Software*, *48*(2), 1-36. <http://www.jstatsoft.org/v48/i02/>.

Schultze, M. (2017). *Constructing Subtests Using Ant Colony Optimization* [Doctoral Dissertation, Freie Universität Berlin]. Refubium – Repositorium der Freien Universität Berlin. <http://dx.doi.org/10.17169/refubium-622>

Schultze, M. (2019). *stuart: Subtests using algorithmic rummaging techniques* (R package version 0.8.0) [Computer software]. The Comprehensive R Archive Network. <https://CRAN.R-project.org/package=stuart>

Siepsiak, M., Śliwerski, A., & Łukasz Dragan, W. (2020). Development and Psychometric Properties of MisoQuest—A New Self-Report Questionnaire for Misophonia. *International journal of environmental research and public health*, *17*(5), 1797-1811. <https://doi.org/10.3390/ijerph17051797>

Vitoratou, S., Hayes, C., Uglik-Marucha, E., & Gregory, J. (2020). *Selective Sound Sensitivity Syndrome Scale (S-Five): a psychometric tool for assessing misophonia. Summary on three waves of sampling and analysis*. PsyArXiv. <https://doi.org/10.31234/osf.io/4dzqn>

White, H. (1980). A Heteroskedasticity-Consistent Covariance Matrix Estimator and a Direct Test for Heteroskedasticity. *Econometrica*, *48*(4), 817-838. <https://doi.org/10.2307/1912934>

**Appendix**

**Table 1**

*Definitions of the Sound Classes of the BMQ-R*

| Sound Class | Definition |
| --- | --- |
| People Eating | Sounds that directly originate from people who are eating. The content-related significance of the eating process and the food is the defining factor. Sounds that fall under this class are, e.g., eating, smacking and chewing in general and in connection with certain foods (e.g., chips, chewing gum, apples, etc.). Other categories include swallowing (if not exclusively significant during eating), environmental sounds typically occurring during eating (e.g., glasses clinking, cutlery rattling, etc.). |
| People Drinking | Sounds that occur directly when drinking liquids. The content-related meaning of the drinking process and the drink is the defining factor. Typical sounds are, e.g., slurping, drinking certain liquids and gurgling while drinking. Other categories include swallowing (if not exclusively significant during drinking), environmental sounds that typically occur during drinking (e.g., clinking of glasses, putting down cups/glasses, spilling of liquids, etc.). |
| Throat and Mouth Sounds | Sounds that are directly caused by the throat, pharynx or mouth of people and do not already include drinking, breathing, eating or human voices. This distinction is due to the experience of a difference in meaning for affected individuals. Typical sounds are, e.g., swallowing (unless it is experienced as exclusively disturbing when eating or drinking), dry mouth, throat clearing, snoring, coughing, kissing, hiccups, etc. |
| Human Voices | Sounds in which human voices can be directly heard and which are not already covered by other categories in terms of content. Typical sounds are, e.g., baby crying, babble of voices, single letters or words, humming, etc. |
| External Environment Sounds | Sounds that occur in the outdoor environment (typically in the city). This includes all sounds that do not already fall under another overlapping category (e.g., rustling, scratching, and squeaking of (everyday) objects or animal noises). Typical noises are, e.g., construction noise, traffic (e.g., cars, trains), thunder, construction equipment (e.g., hammer, saw, lawn mower), balls, etc. |

**Table 1** (continued)

| Sound Class | Definition |
| --- | --- |
| Internal Environment Sounds | Sounds that occur in the environment inside buildings (typically at home). This includes all sounds that do not already fall under another overlapping category (e.g., rustling, scratching, and squeaking of (everyday) objects). Typical noises are e.g., electronic devices (e.g., refrigerator, dishwasher), pen clicking, music, TV noises, keyboard tapping, furniture moving, clock ticking, etc. |
| Nasal Sounds | Sounds that are directly caused by people's noses and where the breathing process is not significant. Typical sounds are, e.g., snorting, sniffing, and sneezing. |
| Finger and Hand Sounds | Sounds that are directly caused by fingers and hands and cannot be assigned to the other classes in terms of content. Typical sounds are, e.g., cracking fingers, cutting fingernails, drumming fingers, clapping hands, rubbing hands. |
| Foot Sounds | Sounds that are directly caused by feet. Typical sounds are, e.g., shuffling feet, footsteps, climbing stairs, flip flops. |
| Rustling, Scratching, and Squeaking | Secondary category of sounds that explicitly include rustling, scratching or squeaking as sound quality. This can sometimes overlap strongly with other categories (e.g., internal or external environmental sounds). The differentiating aspect here is explicitly the sound quality, which is different from other environmental sounds. Typical sounds are, e.g., rustling of paper, plastic or leaves, squeaking or scratching of cutlery or metal. |
| Breathing | Sounds that are directly produced by the human breathing process. Typical sounds are, e.g., breathing through the mouth, exhaling, repetitive and nasal breathing. |
| Animal Sounds | Sounds that directly originate from animals, regardless of the sound quality. The significance of the animal in terms of content is the defining factor. |
| Internal Body Sounds | Secondary category of sounds that originate from organs and which other people often cannot hear. Typical sounds are, e.g., tinnitus, heartbeat, stomach growling. |

**Table 2**

*Results of Confirmatory Factor Analyses and Reliability Estimates for the Pretest Models*

|  | Goodness of fit statistics | | | | | | | | | | |
| --- | --- | --- | --- | --- | --- | --- | --- | --- | --- | --- | --- |
| Model | $\text{χ}_{\text{r}}^{\text{2}}$ | df | $\frac{\text{χ}_{\text{r}}^{\text{2}}}{\text{df}}$ | rCFI | rTLI | rRMSEA (90%-CI) | SRMR | Mc | ECVI | AIC | BIC |
| Disgust Propensity | 10.36^‍†^ | 9 | 1.15 | .99 | 0.99 | .03 (.00 - .09) | .03 | 0.99 | 0.21 | - | - |
| S-Five-E | 521.64^***^ | 265 | 1.97 | .91 | 0.90 | .07 (.06 - .08) | .06 | 0.52 | 3.21 | - | - |
| MisoQuest 14-Item Version | 166.17^***^ | 77 | 2.16 | .90 | 0.88 | .08 (.06 - .09) | .06 | 0.76 | 1.22 | 7515.42 | 7660.90 |
| MisoQuest 12-Item Version | 100.42^***^ | 54 | 1.86 | .94 | 0.92 | .07 (.05 - .09) | .05 | 0.87 | 0.81 | 6426.62 | 6551.32 |
| STICSA 21 Item Version | 327.68^***^ | 188 | 1.74 | .90 | 0.89 | .06 (.05 - .07) | .06 | 0.70 | 2.08 | 12336.73 | 12556.77 |
| STICSA 18 Item Version | 164.13^*^ | 134 | 1.22 | .97 | 0.97 | .03 (.01 - .05) | .04 | 0.91 | 1.24 | 10666.09 | 10855.18 |
| *Note*. *N* = 227-236. $\text{χ}_{\text{r}}^{\text{2}}$ = robust χ² value; rCFI = robust Comparative Fit Index; rTLI = robust Tucker-Lewis Index; rRMSEA = robust Root Mean Square Error of Approximation; SRMR = Standardized Root Mean Square Residual; Mc = McDonald’s Centrality Index; ECVI = Expected Cross Validation Index; AIC = Akaike Information Criterion; BIC = Bayesian Information Criterion.  ^‍†^n.s. ^*^*p* < .05. ^***^*p* < .001 | | | | | | | | | | | |

**Table 3**

*Reliability Estimates for the Pretest Scales*

| Model/Scale | ω (95%-CI) | |
| --- | --- | --- |
| Disgust Propensity | .75 (.68 - .81) | |
| S-Five-E  External Appraisals | .90 (.87 - .92) | |
| Internal Appraisals | .88 (.85 - .90) | |
| Impact | .87 (.83 - .89) | |
| Outbursts | .83 (.79 - .87) | |
| Threat | .89 (.81 - .89) | |
| MisoQuest 12 Item Optimized Version | .87 (.82 - .90) | |
| STICSA 18 Item Optimized Version Trait Somatic Anxiety | .82 (.78 - .85) | |
| Trait Cognitive Anxiety | .86 (.82 - .88) | |
| *Note*. *N* = 227 - 236. ω = McDonald’s Omega (1999). 95%-confidence intervals were calculated via bias-corrected and accelerated bootstrapping with a bootstrapping sample size *B* = 1000. | |  |

**Table 4**

*Interrater Reliability and Agreement for Rated Sound Classes*

| Sounds | K.α (95%-CI) | κ (95%-CI) | Agree | h_i_ |
| --- | --- | --- | --- | --- |
| People Eating | .00 (-.08- .08) | .37 (.27- .46) | 64.25 | .80 |
| People Drinking | .05 (.01- .09) | .23 (.18- .29) | 29.95 | .62 |
| Throat and Mouth Sounds | .06 (.03- .09) | .19 (.13- .25) | 34.78 | .68 |
| Human Voices | .00 (-.06- .07) | .28 (.20-.37) | 59.90 | .82 |
| External Environment Sounds | .05 (-.05- .15) | .23 (.11-.34) | 64.73 | .86 |
| Internal Environment Sounds | .15 (.12- .19) | .17 (.12-.21) | 13.04 | .50 |
| Nasal Sounds | .04 (.00- .08) | .32 (.26-.38) | 39.20 | .66 |
| Finger and Hand Sounds | .01 (.06- .0) | .25 (.16-.34) | 61.35 | .83 |
| Foot Sounds | .06 (.02- .10) | .23 (.16-.30) | 39.61 | .71 |
| Rustling, Scratching, and Squeaking | .18 (.15- .21) | .09 (.05-.12) | 4.35 | .36 |
| Breathing | .13 (.10- .17) | .17 (.12-.22) | 17.87 | .55 |
| Animal Sounds | .11 (.05- .18) | .30 (.22-.39) | 51.69 | .77 |
| Mean | .07 | .18 | 39.90 | .68 |
| *Note*. *N_Rater_* = 207, *N_Categories_* = 12. K.α = Krippendorff’s α; κ = Conger’s κ; Agree = Simple Percentage Agreement without tolerance; h_i_ = relative frequency of intended classifications.  95%-confidence intervals were calculated via bias-corrected and accelerated bootrapping with a bootstrapping sample size *B* = 1000. | | | | |

1. The index was computed by assessing the number of consecutive items with similar responses (Meade & Craig, 2012). [↑](#footnote-ref-1)
