# Supplementary material for: The Berlin Misophonia Questionnaire Revised (BMQ-R): Development and validation of a symptom-oriented diagnostical instrument for the measurement of misophonia
Source: PLoS One. 2022 Jun 21;17(6):e0269428. doi: 10.1371/journal.pone.0269428 (PMC9212156; doi:10.1371/journal.pone.0269428)
Supplement: S1 Table — (DOCX) [file pone.0269428.s002.docx]

**S1 Table.** **Descriptive Statistics and Psychometric Properties of the BMQ-R Items**

| BMQ-R Item | N^a^ | M | SD | P_i_ | λ_std_ |
| --- | --- | --- | --- | --- | --- |
| General Sound Intolerance Symptoms (GI) | 952 | (4.23) | (0.87) | (90.19) | (.77) |
| GI01. There are sounds that I find emotionally stressful. | 952 | 4.23 | 1.21 | 84.66 | .72 |
| GI02. There are sounds that I find unbearable. | 952 | 4.45 | 1.01 | 89.05 | .73 |
| GI03. There are sounds that I hate much more than other sounds. | 951 | 4.64 | 0.83 | 92.72 | .81 |
| GI04. There are sounds that annoy me extremely. | 952 | 4.72 | 0.73 | 94.33 | .82 |
| Anger Reaction: Presence (pAR) | 789 | (3.34) | (1.65) | (66.91) | (.87) |
| pAR01. These sounds make me furious. ^f^ | 822 | 3.75 | 1.56 | 75.01 | .88 |
| pAR02. When I hear these sounds, I am outraged. ^f^ | 822 | 3.88 | 1.40 | 77.66 | .88 |
| pAR03. I have violent thoughts towards the object or person causing the sounds. | 821 | 2.79 | 1.82 | 55.83 | .81 |
| pAR04. These sounds make me more angry than almost anything in my life. | 824 | 2.96 | 1.81 | 59.13 | .91 |
| Irritation Reaction: Presence (pIR) | 789 | (3.27) | (0.98) | (76.37) | (.76) |
| pIR01. When I hear these sounds, I am immediately irritated. | 824 | 3.27 | 1.73 | 65.36 | .65 |
| pIR02. As soon as I hear these sounds, I get frustrated. | 823 | 3.05 | 1.70 | 61.00 | .73 |
| pIR03. I am extremely sensitive to these sounds. | 821 | 4.36 | 1.13 | 87.16 | .78 |
| pIR04. I feel disturbed by the sounds. | 820 | 4.60 | 0.88 | 91.95 | .90 |
| Disgust Reaction: Presence (pDR) | 789 | (2.88) | (1.84) | (51.13) | (.88) |
| pDR01. One can see my disgust when I hear these sounds. ^f^ | 823 | 2.24 | 1.81 | 44.81 | .84 |
| pDR02. I experience disgust when I hear these sounds. | 823 | 2.89 | 1.85 | 57.79 | .87 |
| pDR03. The sounds trigger physical disgust reactions in me. | 819 | 2.02 | 1.81 | 40.34 | .90 |
| pDR04. I am repulsed by hearing these sounds. | 819 | 3.08 | 1.88 | 61.56 | .92 |
| Anxiety Reaction: Presence (pAX) | 789 | (1.52) | (1.41) | (30.16) | (.85) |
| pAX01. When these sounds occur, I feel threatened. | 820 | 1.52 | 1.74 | 30.46 | .85 |
| pAX02. These sounds cause thoughts that scare me. | 821 | 1.56 | 1.76 | 31.21 | .83 |
| pAX03. I am afraid of these sounds. | 819 | 1.50 | 1.75 | 30.04 | .86 |
| pAX03. Sometimes I feel intense fear or panic in response to these sounds. | 819 | 1.45 | 1.78 | 28.94 | .85 |
| Physical Reaction: Presence (pPR) | 789 | (2.92) | (1.74) | (58.51) | (.90) |
| pPR01. My heart is racing when I hear these sounds. ^f^ | 821 | 2.57 | 1.81 | 51.40 | .92 |
| pPR02. I feel physical pressure (e.g., in my chest) when I hear these sounds. | 821 | 2.38 | 1.89 | 47.65 | .85 |
| pPR03. I am physically very tense with these sounds. ^f^ | 821 | 3.82 | 1.52 | 76.47 | .92 |
| Anger Reaction: Anticipation (aAR) | 611 | (2.67) | (1.72) | (53.89) | (.95) |
| aAR01. The imagination of these sounds makes me angry. | 621 | 2.67 | 1.86 | 53.37 | .95 |
| aAR02. The thought of these sounds makes me aggressive. ^f^ | 619 | 2.72 | 1.83 | 54.41 | .95 |
| Irritation Reaction: Anticipation (aIR) | 611 | (2.95) | (1.82) | (56.15) | (.91) |
| aIR01. I am annoyed by the imagination of the sounds. | 618 | 2.96 | 1.79 | 59.19 | .89 |
| aIR02. When I imagine these sounds, I feel disturbed. | 621 | 2.66 | 1.88 | 53.11 | .93 |
| Disgust Reaction: Anticipation (aDR) | 611 | (2.02) | (1.62) | (35.88) | (.91) |
| aDR01. When I imagine that I hear these sounds, I am already disgusted. | 620 | 2.02 | 1.86 | 40.35 | .91 |
| aDR02. One can see my disgust when I only think of the sounds. ^f^ | 621 | 1.57 | 1.65 | 31.40 | .91 |
| Anxiety Reaction: Anticipation (aAX) | 611 | (2.75) | (1.41) | (31.86) | (.93) |
| aAX01. I feel anxious when I think about the next situation with the sounds. | 619 | 1.35 | 1.69 | 27.04 | .89 |
| aAX02. I am afraid of the next situation with the sounds. | 619 | 1.83 | 1.87 | 36.67 | .96 |
| Physical Reaction: Anticipation (aPR) | 611 | (1.35) | (1.60) | (45.96) | (.88) |
| aPR01. When I imagine these sounds, I am physically very tense. ^f^ | 621 | 2.75 | 1.83 | 55.04 | .92 |
| aPR02. My heart races when I imagine the sounds. ^f^ | 621 | 1.84 | 1.80 | 36.88 | .84 |
| Recognition of Excess (RE) | 696 | (1.50) | (1.52) | (55.39) | (.88) |
| RE01. My reaction to these sounds is excessive. ^f^ | 705 | 2.50 | 1.74 | 50.07 | .91 |
| RE02. I react disproportionately emotionally to these sounds. ^f^ | 702 | 3.15 | 1.74 | 62.96 | .88 |
| RE03. My reaction to these sounds is extreme. | 704 | 3.10 | 1.77 | 61.93 | .94 |
| RE04. I have such exaggerated thoughts and feelings when I hear these sounds that I feel like I'm crazy. | 705 | 2.33 | 1.94 | 46.61 | .82 |
| Recognition of Disproportionality (RD) | 696 | (3.37) | (1.63) | (67.40) | (.87) |
| RD01. Actually, I should not have to react to these sounds like that. | 705 | 4.01 | 1.33 | 80.20 | .72 |
| RD02. I react unreasonably to these sounds. ^f^ | 705 | 3.15 | 1.72 | 63.06 | .94 |
| RD03. My reaction to these sounds is inappropriate. ^f^ | 702 | 3.35 | 1.68 | 66.92 | .93 |
| RD04. It is actually unreasonable to react to these sounds in the way I do. ^f^ | 704 | 2.97 | 1.77 | 59.43 | .90 |
| General Dysregulation (GD) | 708 | (2.97) | (1.22) | (54.55) | (.86) |
| GD01. When I hear these sounds, I feel like I have myself under control. (R) ^f^ | 725 | 2.97 | 1.55 | 59.42 | .82 |
| GD02. I can restrain myself with these sounds. (R) | 726 | 2.60 | 1.48 | 52.01 | .89 |
| GD03. If I want to suppress my reaction to these sounds, I succeed. (R) | 726 | 2.82 | 1.52 | 56.36 | .81 |
| GD04. I can control myself with these sounds. (R) ^f^ | 726 | 2.52 | 1.48 | 50.41 | .90 |
| Behavioral Dysregulation (BD) | 708 | (2.79) | (1.62) | (44.06) | (.78) |
| BD01. Sometimes I unintentionally "freak out" at these sounds. ^f^ | 726 | 2.79 | 1.86 | 55.84 | .91 |
| BD02. I do things because of the sounds, which I later regret. ^f^ | 726 | 2.07 | 1.79 | 41.35 | .87 |
| BD03. I cannot help but imitate these sounds. | 725 | 0.94 | 1.44 | 18.76 | .53 |
| BD04. Unintentionally, bad comments about these sounds burst out of me. | 727 | 3.02 | 1.73 | 60.30 | .81 |
| Cognitive Dysregulation (CD) | 708 | (3.83) | (1.16) | (74.55) | (.84) |
| CD01. When I hear these sounds, I can only concentrate on the sounds. | 725 | 3.83 | 1.35 | 76.58 | .88 |
| CD02. When I hear these sounds, I cannot think of anything else. | 726 | 3.58 | 1.47 | 71.65 | .92 |
| CD03. Nothing can distract me from the sounds anymore when I hear them. | 726 | 3.44 | 1.51 | 68.79 | .84 |
| CD04. I manage to block out these noises. (R) | 726 | 4.06 | 1.25 | 81.18 | .74 |
| Emotional Dysregulation (ED) | 708 | (3.18) | (0.91) | (54.64) | (.83) |
| ED01. My feelings get completely out of control with the sounds. | 724 | 2.75 | 1.78 | 54.92 | .92 |
| ED02. I find it difficult to bear these sounds emotionally. ^f^ | 724 | 3.42 | 1.69 | 68.40 | .88 |
| ED03. I am so emotionally overwhelmed by the sounds that I burst into tears. | 724 | 1.24 | 1.60 | 24.81 | .69 |
| ED04. If I cannot avoid these sounds, I become emotionally over-sensitive. ^f^ | 724 | 3.52 | 1.65 | 70.41 | .85 |
| Reactive Avoidance Behavior (rAB) | 662 | (3.41) | (1.66) | (68.15) | (.70) |
| rAB01. I feel a strong urge to avoid situations with the sounds. ^f^ | 667 | 3.86 | 1.54 | 77.15 | .93 |
| rAB02. I immediately cover my ears when I hear these sounds. | 668 | 2.55 | 1.82 | 51.08 | .53 |
| rAB03. I try to drown out or attenuate the stressful sounds with pleasant sounds. | 668 | 3.17 | 1.86 | 63.44 | .50 |
| rAB04. When I hear these sounds, I immediately think of getting away from the source of the sounds. | 667 | 4.05 | 1.41 | 80.93 | .83 |
| Anticipatory Avoidance Behavior (aAB) | 662 | (1.69) | (1.51) | (53.89) | (.90) |
| aAB01. In order not to have to hear these noises I prefer to be alone. ^f^ | 668 | 2.70 | 2.00 | 53.90 | .81 |
| aAB02. I avoid situations in which I expect these sounds. | 667 | 2.94 | 1.82 | 58.74 | .94 |
| aAB03. I actively avoid certain places where I expect these sounds. ^f^ | 667 | 2.63 | 1.90 | 52.53 | .94 |
| aAB04. If I expect the sounds somewhere, I do not go there. | 666 | 2.52 | 1.82 | 50.39 | .92 |
| Distress (DS) | 640 | (3.82) | (1.38) | (62.50) | (.90) |
| DS01. I am often under stress because of the sounds. ^f^ | 648 | 3.39 | 1.83 | 67.87 | .95 |
| DS02. I am depressed because of my reaction to these sounds. | 651 | 2.13 | 1.90 | 42.67 | .94 |
| DS03. These sounds often put me in a negative mood. ^f^ | 649 | 3.18 | 1.81 | 63.67 | .83 |
| DS04. I feel strongly emotionally burdened because of these sounds. | 651 | 3.08 | 1.86 | 61.69 | .87 |
| DS05. I am bothered by these noises. | 649 | 3.83 | 1.59 | 76.58 | .92 |
| Functional Impairment (FI) | 640 | (2.32) | (1.79) | (33.19) | (.82) |
| FI01. My life is severely affected by these noises. ^f^ | 651 | 1.84 | 1.81 | 36.77 | .93 |
| FI02. Because of the sounds, I was already seriously considering changing my job or school. | 651 | 0.96 | 1.64 | 19.26 | .70 |
| FI03. Living together in my family is considerably impaired because of the sounds. | 649 | 1.82 | 1.86 | 36.49 | .84 |
| FI04. My life is determined by the sounds. | 650 | 1.39 | 1.68 | 27.75 | .89 |
| FI05. I find it difficult to have an everyday conversation because of the sounds. | 650 | 1.40 | 1.68 | 28.00 | .64 |
| FI06. These sounds negatively affect my everyday life and my life. ^f^ | 650 | 2.32 | 1.93 | 46.43 | .96 |
| FI07. My response to these sounds has negative consequences for me. ^f^ | 650 | 1.45 | 1.87 | 37.63 | .79 |

^a^ Values for the factors reflect the sample size for the estimation of the respective factor model. (R) = item needs to be recoded. P_i_ = item difficulty; λ_std_ = completely standardized factor loadings as defined in the respective correlated factor models. Unstandardized means and standard deviations of the coefficients per factor are in parentheses. Original items were naïvely translated from German.
^f^ The item was also incorporated in the original BMQ.
