# Supplementary material for: The Berlin Misophonia Questionnaire Revised (BMQ-R): Development and validation of a symptom-oriented diagnostical instrument for the measurement of misophonia
Source: PLoS One. 2022 Jun 21;17(6):e0269428. doi: 10.1371/journal.pone.0269428 (PMC9212156; doi:10.1371/journal.pone.0269428)
Supplement: S2 Table — (DOCX) [file pone.0269428.s003.docx]

**S2 Table. Analyses of the Dimensionality of Highly Correlated BMQ-R Symptom-Scales**

|  | Goodness of fit statistics | | | | | | | | |
| --- | --- | --- | --- | --- | --- | --- | --- | --- | --- |
| Model | χ²_s_(df) | CFI_s_ | RMSEA_s_ (90% CI) | SRMR | Model Comp. | Δχ²_s_(Δdf) | ΔCFI_s_ | ΔRMSEA_s_ | ΔSRMR |
| M1: Anger Reaction Presence and Irritation Reaction Presence – Two Factor Model | 119.01^***^ (19) | .99 | .09 (.08-.11) | .04 | - |  |  |  |  |
| M2: Anger Reaction Presence and Irritation Reaction Presence – One Factor Model | 183.85^***^ (20) | .98 | .12 (.10-.13) | .05 | M1 | 42.77^***^  (1) | .01 | .03 | .01 |
| M3: Irritation Reaction Presence and Physical Reaction Presence – Two Factor Model | 84.73^***^  (13) | .99 | .10 (.08-.11) | .04 | - |  |  |  |  |
| M4: Irritation Reaction Presence and Physical Reaction Presence – One Factor Model | 166.72^***^  (14) | .97 | .13 (.12-.15) | .06 | M3 | 59.60^***^  (1) | .02 | .03 | .02 |
| M5: Disgust Reaction Presence and Disgust Reaction Anticipation – Two Factor Model | 58.22^***^  (8) | 1.00 | .10 (.08-.13) | .02 | - |  |  |  |  |
| M6: Disgust Reaction Presence and Disgust Reaction Anticipation – One Factor Model | 160.17^***^ (9) | .99 | .17 (.14-.19) | .04 | M5 | 55.77^***^ (1) | .01 | .07 | .02 |
| M7: Anxiety Reaction Presence and Physical Reaction Presence – Two Factor Model | 18.68^†^  (13) | 1.00 | .03 (.00-.05) | .02 | - |  |  |  |  |
| M8: Anxiety Reaction Presence and Physical Reaction Presence – One Factor Model | 182.63^***^  (14) | .98 | .14 (.12-.16) | .06 | M7 | 72.98^***^  (1) | .02 | .11 | .04 |
| M9: Anxiety Reaction Presence and Anxiety Reaction Anticipation – Two Factor Model | 31.91^***^ (8) | 1.00 | .07 (.05-.10) | .02 | - |  |  |  |  |
| M10: Anxiety Reaction Presence and Anxiety Reaction Anticipation – One Factor Model | 174.04^***^ (9) | .98 | .17 (.15-.20) | .04 | M9 | 71.62^***^ (1) | .02 | .10 | .02 |
| M11: Physical Reaction Presence and Physical Reaction Anticipation – Two Factor Model | 39.00^***^ (4) | .99 | .12 (.09-.15) | .03 | - |  |  |  |  |
| M12: Physical Reaction Presence and Physical Reaction Anticipation – One Factor Model | 123.53^***^ (5) | .98 | .20 (.17-.23) | .05 | M11 | 57.49^***^ (1) | .01 | .08 | .02 |
| Model | χ²_s_(df) | CFI_s_ | RMSEA_s_ (90% CI) | SRMR | Model Comp. | Δχ²_s_(Δdf) | ΔCFI_s_ | ΔRMSEA_s_ | ΔSRMR |
| M13: Anger Reaction Anticipation and Irritation Reaction Anticipation – Two Factor Model | 0.12^†^ (1) | 1.00 | .00 (.00-.08) | .00 | - |  |  |  |  |
| M14: Anger Reaction Anticipation and Irritation Reaction Anticipation – One Factor Model | 156.04^***^ (2) | .99 | .35 (.31-.40) | .06 | M13 | 110.37^***^ (1) | .01 | .35 | .06 |
| M15: Anger Reaction Anticipation and Physical Reaction Anticipation – Two Factor Model | 2.73^†^ (1) | 1.00 | .05 (.00-.13) | .00 | - |  |  |  |  |
| M16: Anger Reaction Anticipation and Physical Reaction Anticipation – One Factor Model | 117.77^***^ (2) | .99 | .31 (.26-.35) | .05 | M15 | 83.29^***^ (1) | .01 | .26 | .05 |
| M17: Irritation Reaction Anticipation and Physical Reaction Anticipation – Two Factor Model | 0.66^†^ (1) | 1.00 | .00 (.00-.10) | .00 | - |  |  |  |  |
| M18: Irritation Reaction Anticipation and Physical Reaction Anticipation – One Factor Model | 103.93^***^ (2) | .98 | .29 (.24-.34) | .05 | M17 | 77.18^***^ (1) | .02 | .29 | .05 |
| M19: Recognition of Disproportionality and Recognition of Excess – Two Factor Model | 73.70^***^ (19) | 1.00 | .07 (.05-.09) | .02 | - |  |  |  |  |
| M20: Recognition of Disproportionality and Recognition of Excess – One Factor Model | 530.84^***^ (20) | .97 | .20 (.19-.22) | .07 | M19 | 136.47^***^ (1) | .03 | .13 | .05 |
| M21: Recognition of Excess and Anger Reaction Presence – Two Factor Model | 93.12^***^ (19) | .99 | .08 (.06-.10) | .02 | - |  |  |  |  |
| M22: Recognition of Excess and Anger Reaction Presence – One Factor Model | 452.54^***^ (20) | .97 | .19 (.17-.20) | .07 | M21 | 114.34^***^ (1) | .02 | .09 | .05 |
| M23: Recognition of Excess and Irritation Reaction Presence – Two Factor Model | 92.23^***^ (19) | .99 | .08 (.06-.10) | .04 | - |  |  |  |  |
| M24: Recognition of Excess and Irritation Reaction Presence – One Factor Model | 273.06^***^ (20) | .98 | .14 (.13-.16) | .06 | M23 | 80.16^***^ (1) | .01 | .06 | .02 |
| M25: Recognition of Excess and Emotional Dysregulation – Two Factor Model | 107.34^***^ (19) | .99 | .09 (.07-.10) | .02 | - |  |  |  |  |
| M26: Recognition of Excess and Emotional Dysregulation – One Factor Model | 194.77^***^ (20) | .99 | .12 (.10-.13) | .04 | M25 | 65.39^***^ (1) | .00 | .03 | .02 |
| Model | χ²_s_(df) | CFI_s_ | RMSEA_s_ (90% CI) | SRMR | Model Comp. | Δχ²_s_(Δdf) | ΔCFI_s_ | ΔRMSEA_s_ | ΔSRMR |
| M27: Recognition of Excess and Distress – Two Factor Model | 140.65^***^ (26) | .99 | .08 (.07-.10) | .03 | - |  |  |  |  |
| M28: Recognition of Excess and Distress – One Factor Model | 453.04^***^ (27) | .98 | .16 (.15-.17) | .06 | M27 | 92.93^***^ (1) | .01 | .08 | .03 |
| M29: Cognitive Dysregulation and Irritation Reaction Presence – Two Factor Model | 76.72^***^ (19) | .99 | .07 (.05-.09) | .03 | - |  |  |  |  |
| M30: Cognitive Dysregulation and Irritation Reaction Presence – One Factor Model | 211.88^***^ (20) | .97 | .13 (.11-.14) | .05 | M29 | 73.34^***^ (1) | .02 | .06 | .02 |
| M31: Cognitive Dysregulation and Emotional Dysregulation – Two Factor Model | 54.80^***^ (19) | 1.00 | .06 (.04-.07) | .02 | - |  |  |  |  |
| M32: Cognitive Dysregulation and Emotional Dysregulation – One Factor Model | 389.93^***^ (20) | .96 | .17 (.16-.19) | .07 | M31 | 112.05^***^ (1) | .04 | .11 | .05 |
| M33: Emotional Dysregulation and Anger Reaction Presence – Two Factor Model | 58.64^***^ (19) | 1.00 | .06 (.04-.08) | .02 | - |  |  |  |  |
| M34: Emotional Dysregulation and Anger Reaction Presence – One Factor Model | 359.23^***^ (20) | .97 | .17 (.15-.18) | .07 | M33 | 105.34^***^ (1) | .03 | .11 | .05 |
| M35: Emotional Dysregulation and Irritation Reaction Presence – Two Factor Model | 69.36^***^ (19) | .99 | .07 (.05-.08) | .04 | - |  |  |  |  |
| M36: Emotional Dysregulation and Irritation Reaction Presence – One Factor Model | 147.78^***^ (20) | .98 | .10 (.09-.12) | .05 | M35 | 50.46^***^ (1) | .01 | .03 | .01 |
| M37: Emotional Dysregulation and Physical Reaction Presence – Two Factor Model | 34.12^***^ (13) | 1.00 | .05 (.03-.07) | .02 | - |  |  |  |  |
| M38: Emotional Dysregulation and Physical Reaction Presence – One Factor Model | 171.37^***^ (14) | .98 | .14 (.12-.15) | .04 | M37 | 76.05^***^ (1) | .02 | .09 | .02~~4~~ |

| Model | χ²_s_(df) | CFI_s_ | RMSEA_s_ (90% CI) | SRMR | Model Comp. | Δχ²_s_(Δdf) | ΔCFI_s_ | ΔRMSEA_s_ | ΔSRMR |
| --- | --- | --- | --- | --- | --- | --- | --- | --- | --- |
| M39: Emotional Dysregulation and Distress – Two Factor Model | 105.64^***^ (26) | 1.00 | .07 (.06-.09) | .02 | - |  |  |  |  |
| M40: Emotional Dysregulation and Distress – One Factor Model | 311.31^***^ (27) | .99 | .13 (.12-.14) | .05 | M39 | 80.78^***^ (1) | .01 | .06 | .03 |
| M41: Emotional Dysregulation and Functional Impairment – Two Factor Model | 80.64^***^ (43) | 1.00 | .04 (.02-.05) | .02 | - |  |  |  |  |
| M42: Emotional Dysregulation and Functional Impairment – One Factor Model | 604.25^***^ (44) | .97 | .14 (.13-.15) | .07 | M41 | 133.07^***^ (1) | .03 | .10~~1~~ | .05 |
| M43: Emotional Dysregulation and General Sound Intolerance Symptoms – Two Factor Model | 96.06^***^ (19) | .99 | .08 (.07-.10) | .04 | - |  |  |  |  |
| M44: Emotional Dysregulation and General Sound Intolerance Symptoms – One Factor Model | 242.24^***^ (20) | .98 | .13 (.12-.15) | .08 | M43 | 60.59^***^ (1) | .01 | .05 | .04 |
| M45: Reactive Avoidance and Anticipatory Avoidance – Two Factor Model | 54.83^***^ (19) | 1.00 | .06 (.04-.07) | .03 | - |  |  |  |  |
| M46: Reactive Avoidance and Anticipatory Avoidance – One Factor Model | 272.33^***^ (20) | .98 | .14 (.13-.16) | .06 | M45 | 98.17^***^ (1) | .02 | .08 | .03 |
| M47: Distress and Anger Reaction Presence – Two Factor Model | 37.18^***^ (26) | 1.00 | .03 (.00-.04) | .01 | - |  |  |  |  |
| M48: Distress and Anger Reaction Presence – One Factor Model | 525.42^***^ (27) | .97 | .17 (.16-.19) | .07 | M47 | 121.48^***^ (1) | .03 | .14 | .06 |
| M49: Distress and Irritation Reaction Presence – Two Factor Model | 106.21^***^ (26) | .99 | .07 (.06-.09) | .04 | - |  |  |  |  |
| M50: Distress and Irritation Reaction Presence – One Factor Model | 246.76^***^ (27) | .99 | .12 (.10-.13) | .05 | M49 | 72.92^***^ (1) | .00 | .05 | .01 |
| M51: Distress and Physical Reaction Presence – Two Factor Model | 54.36^***^  (19) | 1.00 | .06 (.04-.07) | .02 | - |  |  |  |  |
| M52: Distress and Physical Reaction Presence – One Factor Model | 267.82^***^  (20) | .99 | .14  (.13-.16) | .05 | M51 | 86.87^***^  (1) | .01 | .08 | .03 |
| Model | χ²_s_(df) | CFI_s_ | RMSEA_s_ (90% CI) | SRMR | Model Comp. | Δχ²_s_(Δdf) | ΔCFI_s_ | ΔRMSEA_s_ | ΔSRMR |
| M53: Distress and Functional Impairment – Two Factor Model | 81.75^*^  (53) | 1.00 | .03 (.02-.04) | .02 | - |  |  |  |  |
| M54: Distress and Functional Impairment – One Factor Model | 302.42^***^  (54) | .99 | .09 (.08-.10) | .03 | M53 | 66.75^***^  (1) | .01 | .06 | .01 |
| M55: Distress and General Sound Intolerance Symptoms – Two Factor Model | 140.95^***^ (26) | .99 | .08 (.07-.10) | .04 | - |  |  |  |  |
| M56: Distress and General Sound Intolerance Symptoms – One Factor Model | 330.59^***^ (27) | .98 | .14 (.12-.15) | .08 | M55 | 68.88^***^ (1) | .01 | .06 | .04 |
| M57: General Sound Intolerance Symptoms and Anger Reaction Presence – Two Factor Model | 44.28^***^ (19) | 1.00 | .05 (.03-.06) | .02 | - |  |  |  |  |
| M58: General Sound Intolerance Symptoms and Anger Reaction Presence – One Factor Model | 220.50^***^ (20) | .98 | .13 (.11-.14) | .06 | M57 | 60.26^***^ (1) | .02 | .08 | .04 |
| M59: General Sound Intolerance Symptoms and Irritation Reaction Presence – Two Factor Model | 94.97^***^ (19) | .99 | .08 (.06-.10) | .04 | - |  |  |  |  |
| M60: General Sound Intolerance Symptoms and Irritation Reaction Presence – One Factor Model | 112.28^***^ (20) | .99 | .09 (.07-.10) | .04 | M59 | 13.03^***^ (1) | .00 | .01 | .00 |

*N* = 609-619. CFI_s_ = scaled Comparative Fit Index; RMSEA_s_ = scaled Root Mean Square Error of Approximation; SRMR = Standardized Root Mean Square Residual. All models were estimated with the WLSMV estimator and indicators were treated as ordered categories. A scaled χ² difference test was used.
^†^n.s. ^*^*p* < .05. ^**^*p* < .01. ^***^*p* < .001.
