# Supplementary material for: The Berlin Misophonia Questionnaire Revised (BMQ-R): Development and validation of a symptom-oriented diagnostical instrument for the measurement of misophonia
Source: PLoS One. 2022 Jun 21;17(6):e0269428. doi: 10.1371/journal.pone.0269428 (PMC9212156; doi:10.1371/journal.pone.0269428)
Supplement: Attachment — Submitted filename: Response to the Reviewers.docx [file pone.0269428.s004.docx]

**Manuscript PONE-D-21-25396**

**Response to the Reviewers**

Dear Dr. Brito-Costa,

Thank you for giving us the opportunity to submit a revised version of our manuscript “The Berlin Misophonia Questionnaire Revised (BMQ-R): Development and validation of a symptom-oriented diagnostical instrument for the measurement of misophonia”. We would also like to thank you and the reviewer for the time and effort you dedicated on giving us feedback. In our revised manuscript, we tried to incorporate every aspect made by the reviewer. Below, you find a response to each of the reviewer’s comments.

1. Is the manuscript technically sound, and do the data support the conclusions?

Reviewer 1: Partly.

Author response: We double-checked our methodological approach, our statistical analyses and the conclusions drawn. We believe that our data analyses support the conclusions made. Could you give us an example on where the manuscript is not technically sound and which conclusions are only partly supported by the analyses?

2. Has the statistical analysis been performed appropriately and rigorously?

Reviewer 1: N/A.

Author response: In the course of double-checking, we also re-calculated all of our statistical analyses and corrected minor errors in the manuscript, which do not affect interpretation or conclusions made before. These numerical errors were mainly based on rounding issues and errors in the specification of the maximum likelihood estimators CFA models. We are confident that our analyses are appropriate and correct.

4. Is the manuscript presented in an intelligible fashion and written in standard English?

Reviewer 1: No.

Author response: We revised the entire manuscript linguistically. To our knowledge, there are no more typographical or grammatical errors.

5. Review Comments to the Author

Reviewer 1: I think this paper is very significant and contemporary and the paper includes important aspects.

Author response: Thank you!

Reviewer 1: […] there are many shortfalls and I do believe this paper needs major correction and almost all of the sub-headings need to rewrite and some sub-heading should be added.

Author response: We would really like to address and correct the raised shortfalls. However, we need to know the specific aspects you have in mind and would therefore like to ask you to specify the detected shortfalls.

Regarding the sub-headings, we reformulated and reorganised mainly those in the theoretical background of the study. While we agree that the headings in the theoretical part indeed needed to be revised, we respectfully disagree regarding the other headings. The idea behind the subheadings labelled as diagnostic criteria/areas is to give a better overview of the complex structure of the questionnaire and to consequently refer to them in the results, too. We argue that this provides an essential orientation and, in our opinion, other headings would not suit to follow the paper, but impair the understanding tremendously.

Reviewer 1: The total length of the article is roughly 70 pages. […] in the case of this article, a considerably shorter length has apparently done less harm than good, where the author can develop his points in full to the due level of sophistication and clarity – an issue to be backed up by evidences articulated thereafter in this decision report

Author response: We agree that the paper is very long and therefore made some major changes to address this issue. In total, we were able to remove five pages and about 1400 words. The final manuscript length is about 12000 words (excluding the abstract, tables, figures, and references). In our opinion, a much shorter article would not meet the requirements for reporting materials and methods and would not allow a full understanding of the test development and comprehensive validation study. A comparable article about a scale development for measuring misophonia, which was recently published in Frontiers, with fewer dimensions and a considerably less extensive validation study is about the same length as our revised article (cf. Rosenthal et al., 2021; Development and Initial Validation of the Duke Misophonia Questionnaire). If you see the potential to further shorten the manuscript, we would appreciate more information on what parts are concerned. Below, you find a description of what we have shortened in the revision.

Firstly, we shortened the introduction so that it concisely describes the current literature and the research gap and purpose of our work. Since our submission in 2021, new instruments have been developed and the article must address the alterations in key literature. That is why we needed to include small new paragraphs, however, we managed to rewrite the remainder, so that the paragraph “Existing instruments for measuring misophonia” is shorter than before. Secondly, we described our new measurement approach and the corresponding hypotheses more concisely. Most of the shortening was done in the methods and results sections. We described the many instruments that we used as short as possible and removed additional information. The CTCM(-1) modelling approach for aversive reactions has been removed. In the discussion, we shortened the paragraph describing model misspecifications and the implications from the CTCM(-1) model. Lastly, we removed the conclusions regarding RDoC and HiTOP.
